# Clinico-pathological study of malignant odontogenic tumours from a national referral centre

**DOI:** 10.1186/s12903-020-01365-3

**Published:** 2021-03-18

**Authors:** Hans Prakash Sathasivam, Chee Lynn Saw, Shin Hin Lau

**Affiliations:** 1 Cancer Research Centre, Institute for Medical Research, National Institute of Health, Ministry of Health Malaysia, Setia Alam, Malaysia; 2 Penang Health Services, Ministry of Health Malaysia, Georgetown, Malaysia

**Keywords:** Odontogenic tumour, Ameloblastic carcinoma, Odontogenic carcinoma, Malignant odontogenic tumour

## Abstract

**Background:**

Malignant odontogenic tumours are extremely rare tumours occurring within the jaws. Our study was performed to determine the demographic and clinico-pathological features of malignant odontogenic tumours amongst a multi-ethnic Asian population.

**Methods:**

This was a retrospective cross-sectional study of malignant odontogenic tumours diagnosed at the Institute for Medical Research, Malaysia, from 2009 to 2019. All cases were independently reviewed and reclassified following the criteria set out in the latest edition of the World Health Organization 2017 reference text. Demographic and clinico-pathological data were recorded for each case.

**Results:**

Twenty-four cases of malignant odontogenic tumours were identified. The patients’ age ranged from 16 to 79 years with the mean age at diagnosis being 50.8 years (SD = 16.18). There was a male predominance (66.7%) in this cohort of patients. The ethnic distribution appeared to reflect the Malaysian population with most cases seen amongst the Malay ethnic group (66.7%). Ameloblastic carcinoma was the most frequently diagnosed malignant odontogenic tumour (45.8%) and was also predominantly seen in males (90.9%). All patients with clear cell odontogenic carcinoma were females. There was no obvious sex predilection in primary odontogenic carcinoma not otherwise specified (NOS). The mandible (79.2%) was more frequently involved compared to the maxilla.

**Conclusions:**

Diagnosis and management of malignant odontogenic tumours are challenging due to the rarity of these tumours. Our study has elucidated the clinico-pathological features of malignant odontogenic tumours seen in a multi-ethnic Asian population.

## Background

Odontogenic tumours (OT) are rare entities derived from epithelial, ectomesenchymal and/or mesenchymal components of the odontogenic (or tooth-forming) apparatus and make up less than 5% of all oral tumours [1, 2]. The majority of OTs are located within bone (intra-osseous) but there are some that occur in the soft tissue overlying tooth-bearing areas (peripheral/extra-osseous) [2].

The classification of odontogenic tumours has always been a much debated topic amongst head and neck pathologists with the nomenclature of specific odontogenic tumours changing over the years [1, 2]. There have even been entities that were classified as tumours (keratocystic odontogenic tumour and calcifying cystic odontogenic tumour) in the previous edition of the World Health Organization (WHO) reference text being re-classified as cysts (odontogenic keratocyst & calcifying odontogenic cyst) in view of their clinical behaviour as well as lack of convincing evidence to classify them as tumours [1, 2]. The latest WHO reference text has also re-introduced “odontogenic carcinosarcoma” that was discarded in the previous edition of the WHO reference text [1, 2]. Better understanding of the molecular profile of odontogenic tumours through a multi-omics approach may eventually enable improved classification of odontogenic tumours [3].

The vast majority of OTs are benign in nature and most studies have found that less than 10% of all odontogenic tumours are malignant [4–14]. However a recent study from Ethiopia on odontogenic tumours found that 19.6% of their cases were MOTs, suggesting ethnic and geographic variation may play a part in the aetiopathogenesis of MOTs [15]. There is very limited data regarding the clinico-pathological features of malignant odontogenic tumours (MOTs). Due to their relative rarity, the majority of information regarding MOTs is mostly based on individual case reports or small case-series or as a minor component of studies focusing on odontogenic tumours. Definitive diagnosis of MOTs may be difficult due to the relative lack of existing information on MOTs. Aside from that, the complex and ever-changing classification of these entities also adds an additional layer of difficulty in studying MOTs. Although there have been advances in molecular studies, diagnosis of MOTs is primarily based on the correlation of clinical, histopathological and radiological findings [1, 2]. MOTs are believed to be locally aggressive with frequent recurrence mandating long-term follow up [4, 16, 17].

The rarity of MOTs highlights the need for additional data to augment the existing literature regarding MOTs to further improve our understanding of these entities. At this point in time, there is limited data on the demographic and clinico-pathological features of MOTs in Malaysia. Our study was performed to add to the existing body of work and also reveal the demographic and clinico-pathological features of MOTs amongst Malaysian patients.

## Methods

This was a cross-sectional study of malignant odontogenic tumours (MOTs) diagnosed at the Stomatology Unit of the Institute for Medical Research (IMR) Malaysia from 2009 until 2019. Cases were excluded if: i) there was incomplete data or; ii) the cases were recurrences. The following data were obtained from the archived records: age at diagnosis; sex; ethnicity; presenting complaint; smoking history; alcohol intake; histological diagnosis; site of tumour and treatment. Data were link-anonymised and recorded into a standardised proforma.

Cases were independently reviewed and classified by two oral & maxillofacial pathologists (blinded to the original diagnosis) following criteria set out in the latest edition of the World Health Organization (WHO) reference text [2]. Briefly, archived haematoxylin and eosin (H&E) stained sections of the cases identified from the database were retrieved and assessed. For cases where the archived H&E stained slides were inadequate for diagnostic interpretation, new sections were cut from the relevant archived formalin-fixed paraffin-embedded (FFPE) blocks and stained with H&E. A consensus meeting was held for discordant and complex cases.

Descriptive statistical analysis was performed using IBM SPSS Statistics for Windows version 26 (IBM Corp., Armonk, N.Y., USA). For continuous data, results were expressed as mean with standard deviation (SD). This study has favourable ethical opinion from the Malaysian Research Ethics Committee (NMRR-19- 673–4734) and complies with Malaysian legislation and guidelines.

## Results

Thirty-two cases were identified from the database. However, eight cases were excluded due to incomplete data leaving only 24 cases for histopathological review. The demographic and clinico-pathologic features of the patients are listed in Table 1.

**Table 1:** Demographic and clinico-pathologic features of patients

Variable	Number of patients
Age at diagnosis; Mean (SD)	50.8 years (16.18)
Sex	
Male	16
Female	8
Ethnicity	
Malay	16
Chinese	3
Indian	2
Iban	1
Bajau	1
Dusun	1
Site	
Maxilla	5
Mandible	19
Diagnosis	
Ameloblastic carcinoma	11
Clear cell odontogenic carcinoma	3
Primary intraosseous carcinoma NOS*	9
Ghost cell odontogenic carcinoma	1

*NOS, not otherwise specified

The age at diagnosis in this cohort ranged from 16 to 79 years of age with the majority (83.3%) being ≥ 40 years old at diagnosis peaking in the 5th & 6th decades of life. The mean age for this group of patients was 50.8 years (SD ± 16.18). The mean age for patients with ameloblastic carcinoma was lower at 47.0 years (SD ± 17.83). There was a male predominance (66.7%) with a 2:1 male to female ratio. The youngest patient was a 16-year old Malay male with ameloblastic carcinoma (AC). All but one patient (95.8%) presented with a complaint of swelling whilst 83.3% of patients also complained of pain associated with the swelling.

Majority of cases involved the mandible (79.2%). Ameloblastic carcinoma was the most frequently diagnosed MOT (45.8%) and was predominantly seen in Malay males; there was only one female patient with AC. Two patients with AC developed pulmonary metastasis. All the clear cell odontogenic carcinomas seen were diagnosed in females. Primary intraosseous carcinoma not otherwise specified (PIOC-NOS) cases appeared to be almost equally distributed between the sexes (Table 2). Two of the PIOC-NOS were believed to have arisen from odontogenic cysts. One case of AC was preceded by an ameloblastoma. There was only one case of ghost cell odontogenic carcinoma. Overall, fifteen patients (62.5%) has a history of smoking, whilst only seven patients (29.2%) had a history of consuming alcoholic beverages. Nine (81.8%) of the AC patients had a history of smoking, whilst only one (9.1%) had a history of consuming alcoholic beverages. Relevant clinico-pathological information for each patient is displayed in Table 2.

**Table 2:** Data of patient with malignant odontogenic tumours (MOTs)

Case	Age range (years)	Ethnicity	Site	Diagnosis	Treatment
1	50–59	Malay	Mandible	Ameloblastic carcinoma	Surgery
2	20–29	Malay	Mandible	Ameloblastic carcinoma	Surgery
3	40–49	Malay	Mandible	Ameloblastic carcinoma	Surgery
4	60–69	Malay	Mandible	Clear cell odontogenic carcinoma	Surgery
5	50–59	Dusun	Maxilla	Primary intraosseous carcinoma NOS	Surgery
6	40–49	Chinese	Mandible	Clear cell odontogenic carcinoma	Surgery
7	50–59	Malay	Mandible	Primary intraosseous carcinoma NOS	Surgery
8	50–59	Indian	Mandible	Primary intraosseous carcinoma NOS	Surgery
9	20–29	Iban	Mandible	Ameloblastic carcinoma	Surgery
10	40–49	Malay	Mandible	Ameloblastic carcinoma	Palliative
11	70–79	Indian	Maxilla	Ameloblastic carcinoma	Palliative
12	40–49	Bajau	Maxilla	Primary intraosseous carcinoma NOS	Surgery
13	40–49	Malay	Maxilla	Primary intraosseous carcinoma NOS	Surgery
14	60–69	Malay	Mandible	Primary intraosseous carcinoma NOS	Surgery
15	50–59	Malay	Mandible	Primary intraosseous carcinoma NOS	Surgery
16	50–59	Malay	Mandible	Ameloblastic carcinoma	Surgery and concurrent chemoradiotherapy
17	70–79	Malay	Maxilla	Clear cell odontogenic carcinoma	Declined
18	60–69	Malay	Mandible	Ameloblastic carcinoma	Declined
19	40–49	Malay	Mandible	Ameloblastic carcinoma	Surgery and radiotherapy
20	10–19	Malay	Mandible	Ameloblastic carcinoma	Surgery
21	40–49	Malay	Mandible	Primary intraosseous carcinoma NOS	Surgery
22	70–79	Chinese	Mandible	Ghost cell odontogenic carcinoma	Surgery
23	50–59	Malay	Mandible	Ameloblastic carcinoma	Surgery
24	20–29	Chinese	Mandible	Primary intraosseous carcinoma NOS	Surgery and radiotherapy

NOS, not otherwise specified

**Fig. 1:**
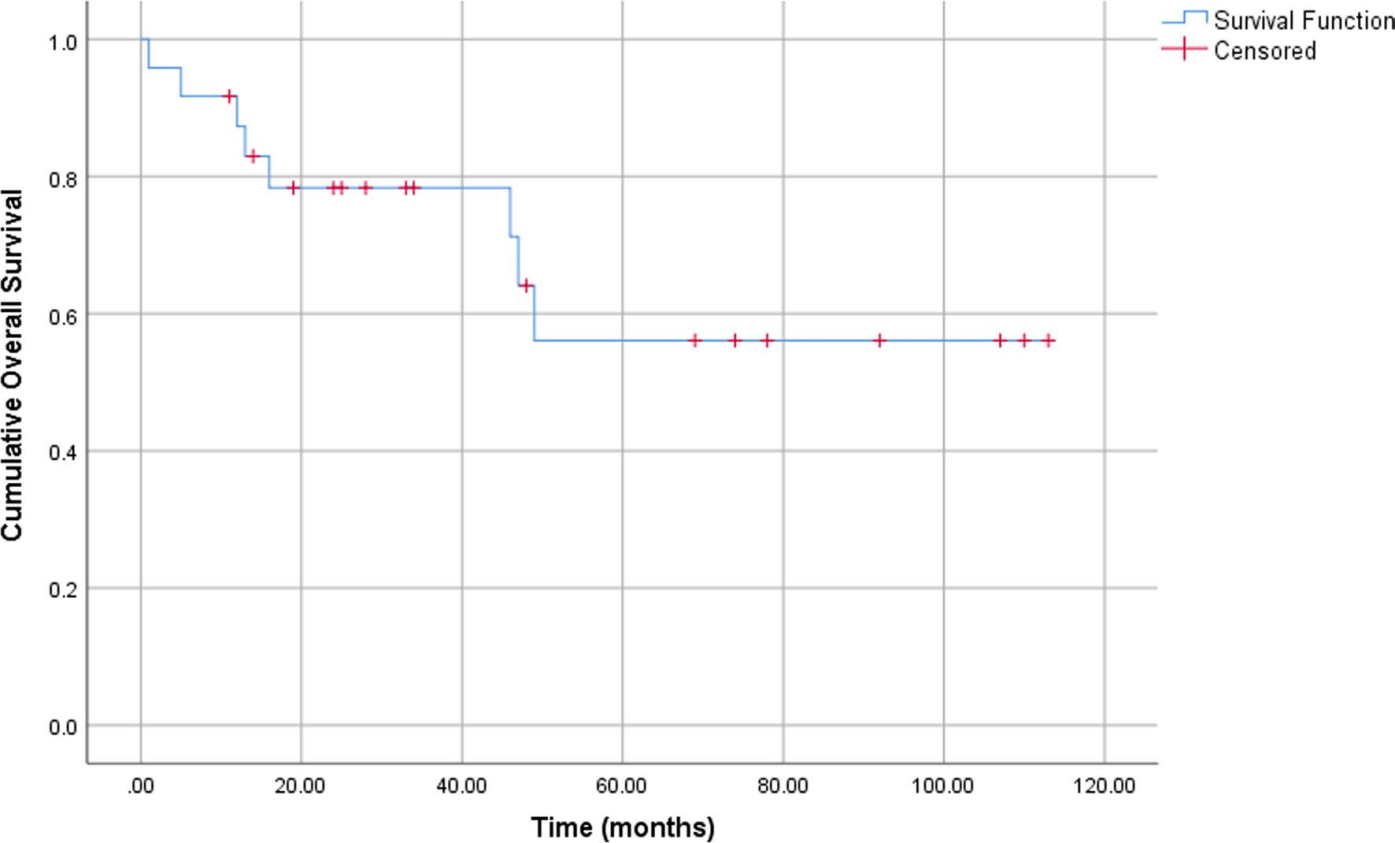
Kaplan–Meier curve for time to event analysis looking at overall survival of patients with MOT

Twenty patients were treated with intent to cure whilst two were treated palliatively and another two declined treatment. Surgery was the primary treatment modality for all cases treated with intent to cure. Two patients received additional post-operative radiotherapy as part of their treatment plan and one received surgery with concurrent chemoradiotherapy. Only sixteen patients (66.7%) were alive and well on  census date. The median follow-up time for these 16 patients was 41 months (inter-quartile range: 64.25). Time to event analysis looking at the overall survival of patients with MOTs is shown in Fig. 1.

## Discussion

Malignant odontogenic tumours are extremely rare. A pooled analysis on the global incidence of odontogenic tumours by Avelar *et. al.* (2011) found that only around 4% of odontogenic tumours were malignant [18]. A recently published Malaysian study on odontogenic tumours made up of 173 cases had only two (1.2%) cases of MOT [5]. Malignant odontogenic tumours can be broadly classified into odontogenic carcinomas, odontogenic sarcomas and odontogenic carcinosarcoma. Although odontogenic carcinosarcomas were re-introduced in the latest edition of the WHO reference text on classification of head and neck tumours, they are extremely rare entities [2]. Over the 11-year period of our study, there were no cases of odontogenic sarcomas or odontogenic carcinosarcomas, highlighting the relative rarity of such lesions compared to odontogenic carcinomas.

Although MOTs can be seen in any age group, the odontogenic carcinomas frequently occur in patients aged > 40 years and this trend is seen in our study as well with more than 80% of cases occurring in those aged ≥ 40 years [2, 12, 15, 18]. The mean age of our patients (50.8 years; SD ± 16.18) was also similar to most other studies focusing on odontogenic carcinomas as there were no sarcomas or carcinosarcomas in our cohort [17, 19, 20]. The mean age for patients with AC was even lower at 47.0 years (SD ± 17.83) similar to the findings of the systematic review by Deng et al. [20]. The male predominance seen in our study is also in line with most other published works, though there are some studies that have reported either no sex predilection or a female preponderance [7–9, 12, 17–19, 21, 22]. Interestingly, when looking at the different MOTs, a striking male preponderance was seen in ACs whilst all the patients diagnosed with CCOC were females. A recent systematic review highlighted that ACs are more frequently seen in males (72.0%) with a male to female ratio of 2.58:1 [20]. The frequent involvement of the mandible in our study is similar to most other studies in the Asian population [7, 17, 20, 23].

The overall ethnic distribution of MOT patients in our study appears to reflect the general ethnic distribution of the Malaysian population, however there does seem to be an overwhelmingly high incidence of ameloblastic carcinoma among Malay patients with only two non-Malay patient having AC. This could be due to genetic predisposition, however further longitudinal studies made up of a larger number of patients is necessary before such conclusions can be made. The role of smoking and alcohol consumption in the aetiopathogenesis of AC is still unclear as these tumours usually arise intra-osseously and are therefore not exposed to carcinogens with perhaps the exception of AC arising from peripheral ameloblastoma. On another note, smoking and alcohol consumption history is usually self-reported and as such the veracity of the information provided itself is fraught with ambiguity [24].

The two most frequently encountered MOTs as reported in the literature are ameloblastic carcinoma (AC) and primary intra-osseous carcinoma NOS (PIOC-NOS) [6, 7, 9, 19, 21, 22]. Ameloblastic carcinoma has been considered to be the malignant counterpart of the ameloblastoma characterized by cytological atypia and the ability to metastasize and is the most frequently encountered MOT. Ameloblastic carcinomas also have been reported to have *BRAF V600E* mutations similar to that seen in ameloblastomas alluding to the possibility that ACs arise from ameloblastomas [25]. Our findings suggest that the majority of ACs arise de novo as only one case in our cohort had a history of pre-existing ameloblastoma. Though some ACs may indeed arise from ameloblastomas it is quite difficult to prove this conclusively and it is currently believed that the large proportion of ACs arise de novo [2]. The previous version of the WHO reference text sub-typed ACs into either; (1) primary type, (2) secondary type (dedifferentiated) intra-osseous or (3) secondary type (dedifferentiated), peripheral. This sub-typing was dropped in the recent edition of the WHO reference text possibly due to the fact that as yet, there is no concrete evidence on the histogenesis of these lesions, the possible role of precursor lesions in the aetiopathogenesis, and the clinical significance of sub-typing [1, 2].

Primary intra-osseous carcinoma NOS (PIOC-NOS) was previously sub-typed either as (1) a solid tumour invading marrow spaces and inducing bone resorption, (2) a squamous carcinoma arising from the lining of an odontogenic cyst or (3) a squamous cancer arising in association with other benign odontogenic tumours [1]. This sub-typing however has been dropped from the latest edition of the WHO reference text possibly due to the uncertainty surrounding the clinical significance of such sub-typing [2]. At this point in time, there is insufficient evidence that sub-typing can reliably predict clinical outcome or overall survival. One difficulty associated with the diagnosis of PIOC-NOS is that once the tumour perforates the cortex and merges with the oral epithelium, distinguishing it from a squamous cell carcinoma arising from the oral mucosa can be challenging especially if there are no obvious odontogenic precursor lesions. Primary intra-osseous carcinoma NOS is also a diagnosis of exclusion having ruled out other carcinomas especially metastatic lesions.

All three clear cell odontogenic carcinomas (CCOC) in our study were female patients which is consistent with pooled findings from other studies [2, 26–28]. One of the differential diagnosis for CCOC is the hyalinizing clear cell carcinoma (HCCC) of the salivary gland which may occur in the jaws [2, 29]. Due to the overlapping clinical, pathological and immunophenotypic patterns, confidently distinguishing between these entities is at times rather difficult. Peripheral palisading in the epithelial islands and the location are thought to be helpful in separating CCOC from HCCC [29, 30]. A large proportion of CCOC have also been shown to have *EWSR1* rearrangements, similar to that found in clear cell salivary gland carcinoma, suggesting that these tumours despite their differing locations, are somewhat related [2, 31, 32].

A major limitation of our study was the inability to obtain complete clinical, radiographic and long-term outcome data for our patients due to the study design and reliance on retrospective data. Very little is known regarding the aetiopathogenesis MOTs with most believed to arise *de-novo* though a proportion of cases also do arise from pre-existing benign lesions such as odontogenic cysts or tumours [2, 33–36]. In our cohort of patients, two cases of PIOC-NOS were believed to have arisen from the epithelial lining of odontogenic cysts and one case of ameloblastic carcinoma was thought to have been preceded by an ameloblastoma. In many cases proving that the malignant tumour originated from a pre-existing benign lesion is extremely difficult. It must be emphasized that diagnosis of malignant odontogenic tumours is challenging; due to its’ rarity and also due to the overlapping features with benign odontogenic tumours as well as other tumours in the maxillofacial region.

## Conclusions

The characterisation, classification and understanding of MOTs will continue to develop as more cases are reported in the literature. This study has added valuable clinico-pathological information on malignant odontogenic tumours that are extremely rare, with scarcity of data in the existing literature especially in the Malaysian setting.

With the advent of molecular profiling, an additional dimension has been added to help further characterise these tumours and perhaps pave the way for improved management of patients with malignant odontogenic tumours.

## Data Availability

The datasets used and/or analysed during the current study are available from the corresponding author on reasonable request.

## Abbreviations

AC: Ameloblastic carcinoma
CCOC: Clear cell odontogenic carcinoma
FFPE: Formalin-fixed paraffin-embedded
H&E: Haematoxylin and eosin
HCCC: Hyalinizing clear cell carcinoma
IMR: Institute for Medical Research
MOT: Malignant odontogenic tumour
NOS: Not otherwise specified
OT: Odontogenic tumour
PIOC-NOS: Primary intraosseous carcinoma not otherwise specified
SD: Standard deviation
WHO: World Health Organization
